# Repetitive afterglow in zirconia by pulsed near-infrared irradiation toward biological temperature sensing

**DOI:** 10.1038/s41598-022-12585-8

**Published:** 2022-05-21

**Authors:** Masaharu Ohashi, Yoshihiro Takahashi, Nobuaki Terakado, Noriko Onoue, Tsuyoshi Shinozaki, Takumi Fujiwara

**Affiliations:** 1 Department of Applied Physics, Graduate School of Engineering, Tohoku University, 6-6-05 Aoba, Aoba-ku, Sendai, Miyagi 980-8579 Japan; 2 Department of Cardiovascular Medicine, National Hospital Organization, Sendai Medical Center, 8-8, 2-chome, Miyagino, Miyagino-ku, Sendai, Miyagi 983-8520 Japan

**Keywords:** Materials for optics, Solid-state chemistry

## Abstract

Photoluminescence provides information about the surrounding environment. In this study, aiming to develop a non-invasive deep body-temperature sensing method, we investigated photoluminescence properties of afterglow zirconia (ZrO_2_) by pulsed near-infrared (NIR) light irradiation based on the biological temperature. Pulsed light irradiation produced optically stimulated luminescence, followed by afterglow, with the property of repeating 100 times or more. Furthermore, the basic principle of temperature measurement was demonstrated through afterglow decay curve measurements. The use of harmless ZrO_2_ as a sensing probe and NIR light, which is relatively permeable to living tissues, is expected to realize temperature measurements in the brain and may also facilitate optogenetic treatment.

## Introduction

Afterglow (AG) is long-lasting photoluminescence (PL) that occurs after photoexcitation and is caused by recombination between holes and electrons trapped at metastable sites and thermally released. It is commonly observed on an emergency sign and watch dial. Trapped electrons can be released by near-infrared (NIR) irradiation as well, i.e., optically stimulated luminescence (OSL). AG and OSL appear to be useful for biological and medical assessments because optical/spectroscopic techniques are considered a non-invasive and contactless approach. For example, Chermont et al. demonstrated bio-imaging using an AG phosphor of Eu^2+^, Dy^3+^, Mn^2+^-tri-doped Ca_0.2_Zn_0.9_Mg_0.9_Si_2_O_6_^1^. In addition, many researchers have focused on developing phosphors that apply to imaging/sensing^2–8^.

Our research group has proposed a new temperature sensing concept for deep parts of the human body based on AG and OSL and then demonstrated a basic principle for temperature determination by the AG decay curve using a chemically-stable and harmless oxide, namely, zirconia (ZrO_2_), as a sensing probe^9^. In addition, external laser stimulation with a wavelength (~ 650–1000 nm) in the NIR region, which has relatively high transmittance to biological tissue (i.e., biological window), enables site-selective and arbitrary-timing measurements, and we have also been able to observe OSL by irradiating a bone sample with a continuous-wave NIR-laser^9^. Meanwhile, continuous irradiation is regarded as an external source of energy in terms of temperature rise and resulting in thermal damage in biological tissue. In this study, to demonstrate biological temperature sensing using AG phosphor, we have investigated the impact of environmental temperature on the PL properties of a ZrO_2_ sample under pulsed NIR irradiation. OSL, along with AG, was observed at 480 nm by repetitive pulsed irradiation, and the observation could be continued over ~ 100 cycles of pulsed irradiation. Furthermore, evaluation of temperature was also demonstrated on the basis of decay curves observed in the AG process.

## Results and discussion

### OSL and AG by pulsed NIR irradiation in ZrO_2_

**Figure 1:**
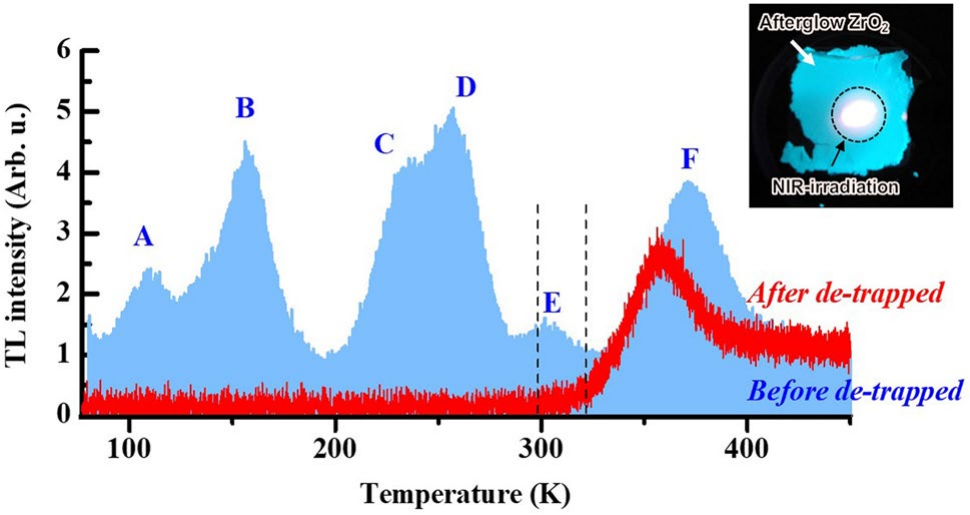
TL spectra in ZrO_2_ samples before and after electron de-trapping at 323 K for 2.5 h. The spectrum before electron de-trapping are refered from ref. 9. The range within the dashed lines indicates the temperature at which AG decay curves were measured. Afterglow ZrO_2_ sample irradiated with a NIR-laser is also shown in the inset. The bright part corresponds to the irradiated area, i.e., OSL.

Figure 1 shows thermoluminescent (TL) spectra of ZrO_2_ sample pre-heated (de-trapped) at 323 K for 2.5 h, along with the result of ZrO_2_ sample not pre-heated. Preparation of the ZrO_2_ sample and its structural analyses are described in Method’s part. According to the TL measurement, thermally treated ZrO_2_ is reported to exhibit several peaks due to several kinds of electron-trapping sites^10–12^. We have also confirmed six primary TL peaks, peaks A–F, with peaks E and F appearing around/above room temperature, attributed to AG and OSL, respectively^9^. The ZrO_2_ sample treated at 1400 °C for 6 h exhibits AG after ultraviolet (UV)-light exposure (254 nm), followed by OSL visually observed at the NIR-laser-irradiated spot (inset). However, the presence of multiple peaks complicates the analysis of AG decay curves^9^. Therefore, prior to the TL measurement, we pre-heated the ZrO_2_ sample at 323 K for 2.5 h to release electrons trapped in trapping sites, appearing below room temperature, i.e., de-trapping. According to the result, peaks A–E vanished, and only peak F at ~ 360 K, which is inactive at biological temperature, was observed in the de-trapped (pre-heated) sample. Hereafter, measurements were performed using the de-trapped sample.

**Figure 2:**
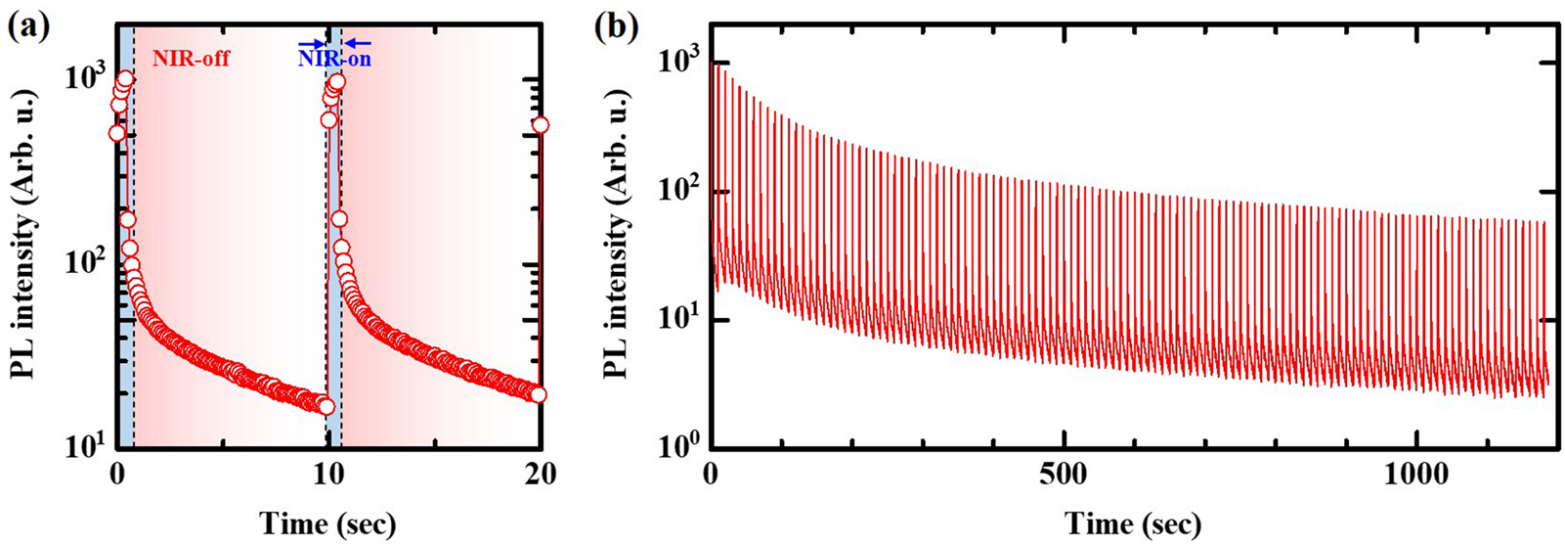
PL intensity as a function of time in ZrO_2_ sample irradiated with a repetitive pulsed NIR light at room temperature: (**a**) Initial period of repetitive irradiation. The blue region corresponds to the width of the pulse and indicates the OSL regime, and the pale-red region indicates the AG regime, i.e., the NIR irradiation stopped. (**b**) Entire measurement period.

Figure 2 shows the PL intensity of the ZrO_2_ sample irradiated with repetitive pulsed NIR light at room temperature. The intensity rapidly increased immediately after irradiation and subsequently decayed (Fig. 2a). The rapid increase in PL intensity is attributed to OSL due to NIR irradiation. The moderate decay after pulsed irradiation is regarded as an AG process because the irradiation was stopped in this term. This leads us to consider that AG decay curves induced by OSL exhibit similar behavior to AG properties previously reported, i.e., the lifetime decreases as the environmental temperature of ZrO_2_ increases^9,13^. Furthermore, we could confirm the rapid PL increase of over 100 times caused by repetitive irradiation (Fig. 2b). On the other hand, the maximum OSL intensity decreased over time (or repetition), e.g., *I*_max;*t*~10_ / *I*_max;*t*~1000_ ~ 0.066,

### Decay curve response to pulsed NIR irradiation and its temperature dependence

Next, we examined the temperature dependence on AG lifetime by pulsed irradiation. Since the release of electrons is caused by thermal excitation, the environmental temperature determines the AG lifetime (or slope of an AG curve). Assuming that AG phosphor contains only one type of electron-trapping site, the temperature dependence on the intensity of AG PL follows a simple exponential relationship:

1 $$\frac{I\left(t\right)}{I\left(0\right)}\propto \mathrm{exp}\left(-at\right),$$

2 $$a=s\cdot \mathrm{exp}\left(-\frac{E}{{k}_{\mathrm{B}}T}\right),$$

where *I*(*t*) represents the PL intensity, *I*(0) represents the initial intensity, *t* represents time, *a* represents the probability of thermal activation of a trapped electron into the conduction band, *E* represents the thermal activation energy of a trapped electron, *s* represents the frequency factor, *k*_B_ represents the Boltzmann constant, and *T* represents temperature. We can obtain the activation energy by observing the temperature dependence on *a,* which corresponds to an inverse of PL lifetime ($1/\tau$). As a result, we can estimate the environmental temperature of phosphor by AG measurements.

**Figure 3:**
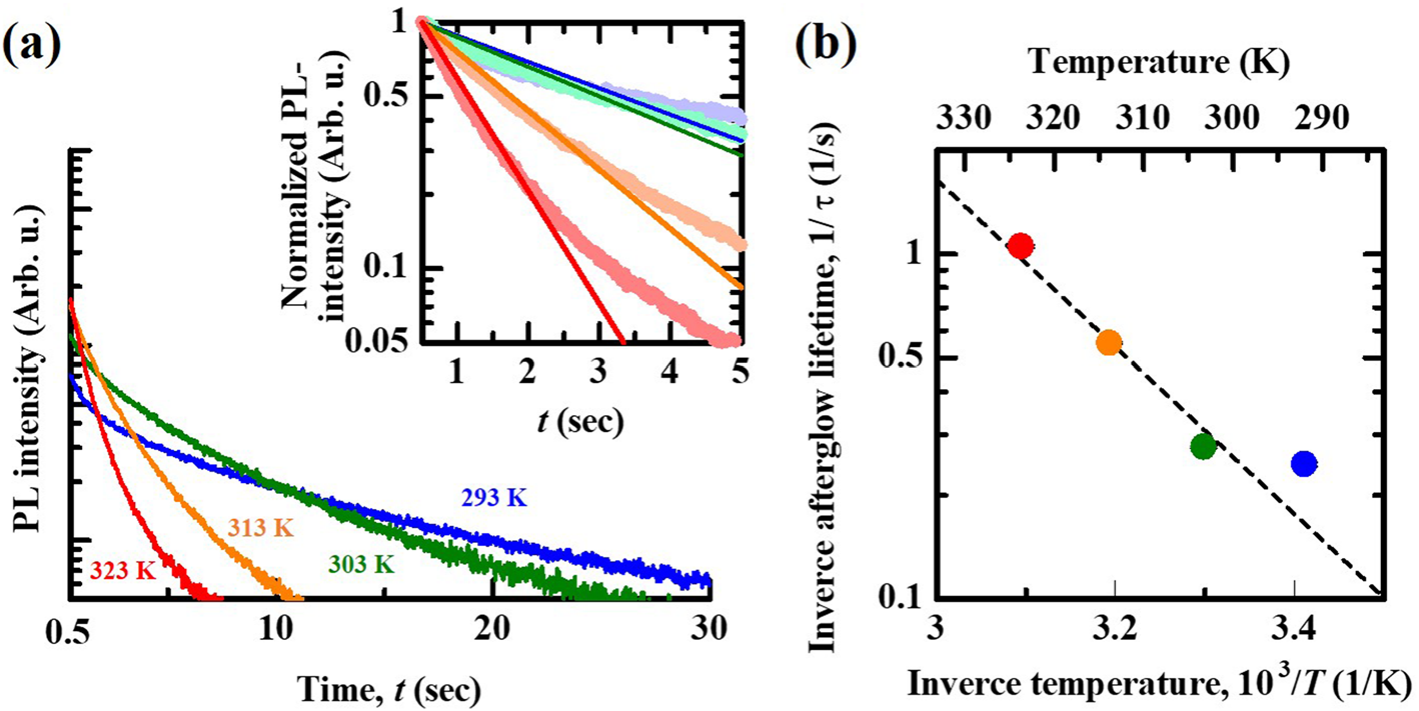
(**a**) Decay curves in the AG regime observed at different temperatures. (**b**) The obtained values of probability as a function of inverse temperature.

Figure 3 shows AG decay curves of ZrO_2_ by single pulsed NIR irradiation at varying environmental temperatures. The decay curves observed at 313 K and 323 K seemed to be monotonically decreased, whereas the initial intensity (*t* <  ~ 1) rapidly decayed and then decreased monotonically (Fig. 3a). This suggests that the OSL intensity partially contributed to the AG intensity; thus, we re-plotted the decay curves, which are normalized by the intensity at *t* = 1 s to evaluate the lifetime by fitting using a single exponential function. As a result, the de-trapped sample tended to exhibit a linear decay response with slopes that increased with increasing temperature. On the other hand, the response at 323 K deviated from the theoretical line (inset), implying that electron release from a deeper trapping site, i.e., peak F, partially contributed to AG decay curves because a trail of peak F is extended around 323 K. The evaluated lifetime and temperature could be plotted according to the Arrhenius approach, resulting in a linear relation (Fig. 3b), yielding activation energy of approximately 0.48 eV. Thus, we demonstrated that temperature can be estimated on the basis of the AG process lifetime by pulsed NIR irradiation simulations.

**Figure 4:**
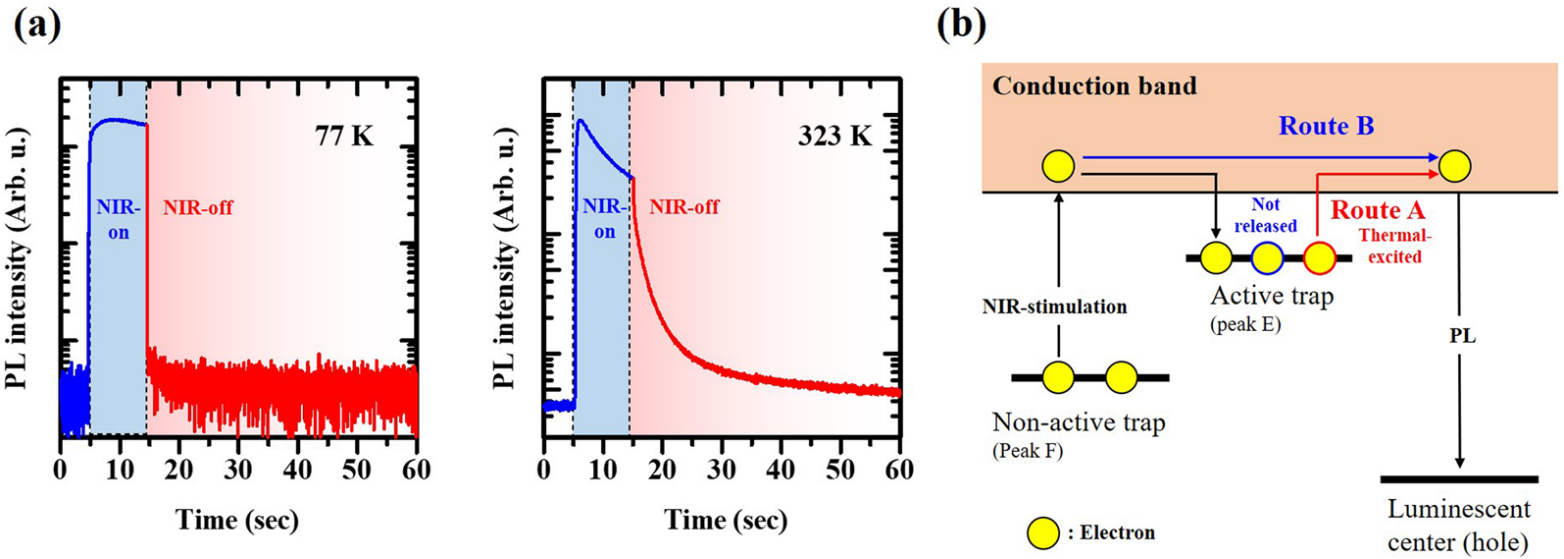
(**a**) PL intensity as a function of time in ZrO_2_ sample irradiated with a single-pulse NIR light at 77 K and 323 K. Blue and red regions indicate OSL and AG regimes, respectively. (**b**) Schematics of the PL process (Valence band is omitted).

The activation energy in this study is comparable to that of AG in ZrO_2_ previously reported^9^. To clarify the AG process in detail, we measured the PL intensity of the de-trapped ZrO_2_ sample through NIR irradiation stimulation at 77 K and 323 K and compared its decay behaviors. Figure 4 depicts PL intensities detected by NIR irradiation with a rectangular waveform (width: 10 s). Immediately after irradiation, obvious PL could be observed at both temperatures (Fig. 4a). At 77 K, the PL is attributed to OSL, which is occurred by the recombination between holes and electrons released by NIR irradiation. In addition, at 323 K, the sample exhibited moderate AG decay when the irradiation was stopped, whereas a rapid decrease in the intensity was observed at 77 K, indicating that AG is barely observed at 77 K. These measurements lead to the conclusion that, at 323 K, electrons trapped in trapping sites, which are inactive at biological temperature (peak F), are released by NIR irradiation, and subsequently, these electrons are partially re-trapped at the thermally active site (peak E) via transition to the conduction band. Eventually, the AG process is observed (route A; Fig. 4b). At 77 K, although electrons released by NIR irradiation are trapped by active sites as well, the thermal release is suppressed because this temperature is significantly lower than peak E and others (route B). According to the result, only OSL can be observed. Therefore, it is reasonable to assume that AG observed immediately after NIR irradiation is regarded as typical AG, and consequently, they are identical. Although the origin of AG in ZrO_2_ is still controversial, Iwasaki et al. suggest that the trapping sites in AG was closely related to the presence of *F*^+^-center (or V_o_^·^ in Kröger-Vink notation) on the basis of measurement of electron spin resonance^13^.

This study can demonstrate the availability of pulsed NIR irradiation, but we should also consider converting the PL wavelength to be compatible with the biological window range. The conversion of PL to a longer wavelength in ZrO_2_ using an appropriate dopant has been reported^14,15^, and a similar study is underway.

## Summary

The temperature dependence of PL properties of ZrO_2_ under pulsed NIR-laser irradiation was investigated for biological temperature sensing applications by decay curve measurements. We demonstrated that environmental temperatures can be estimated from the AG lifetime through the Arrhenius approach. The use of low-toxicity ZrO_2_ as a sensing probe is expected to reduce stress in living organisms. Repetitive pulsed NIR-laser irradiation is expected to not only reduce damage to biological tissue but also shorten the time and improve the accuracy of repetitive temperature measurements.

Non-invasive therapies based on optogenetics have been reported to control memory and sociality via light irradiation of local areas of the brain and neurons, but in these experiments, optical fibers/LEDs are inserted directly into the irradiated areas of mice^16–19^. Therefore, AG and OSL caused by external pulsed NIR irradiation using AG probes may contribute to optogenetic therapy by enabling non-invasive and site-selective optical simulations of local areas.

## Methods

We prepared the ZrO_2_ sample by annealing oxygen-defect activation, and the sample exhibited a broad PL peak at ~ 480 nm due to AG and OSL^9,13^. Therefore, in this study, we also prepared another ZrO_2_ sample by referring to the previous study: Commercial reagent-grade ZrO_2_ powder (purity: 99.9%, Soekawa Chem. Co., Ltd.) was thermally treated at 1400 °C for 6 h to introduce oxygen defects. The heating rate was 10 K/min, and the target temperature was maintained for 6 h in an electric furnace under atmospheric conditions. After the treatment, the ZrO_2_ powder was cooled in the furnace, and finally, the ZrO_2_ sample was obtained.

**Figure 5:**
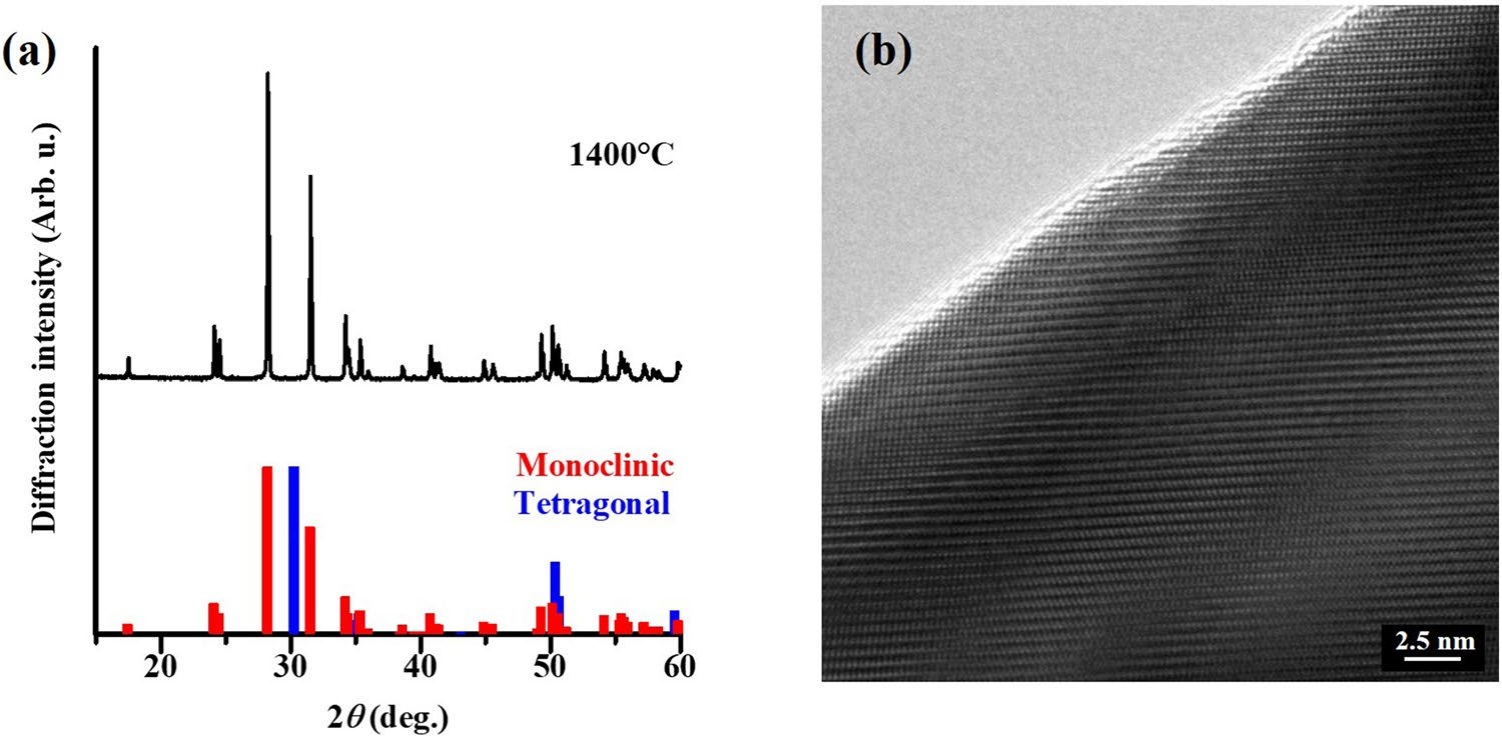
ZrO_2_ powder heat-treated at 1400 °C for 6 h: (**a**) Powder XRD pattern of the sample. ICDD of monoclinic (#36-0420; red bars) and tetragonal ZrO_2_ (#50-1089; blue bars) are also included. (**b**) TEM image.

At room temperature, ZrO_2_ crystallizes in monoclinic system, and undergoes a phase-transition to tetragonal system at ~ 1170 °C. Thermal treatment above the phase-transition temperature introduces oxygen defects, which are associated with the phase transition^13^. Since tetragonal ZrO_2_ has been found to show no PL^20^, structural analysis of the obtained sample was performed by means of an X-ray diffraction (XRD; Cu-Kα). As the result, the phase of treated ZrO_2_ was identified to be monoclinic (Fig. 5a). In addition, a transmission electron microscopy (TEM) revealed clear lattice pattern in the ZrO_2_ grain, suggesting the preservation of crystallinity (Fig. 5b).

The PL intensity as a function of time (decay curve) was measured using a spectrofluorometer with a xenon lamp as the excitation source. The environmental temperature of the ZrO_2_ sample during decay curve measurement was controlled using a cryostat. The PL intensity as a function of temperature (TL spectrum) was also measured using the spectrofluorometer conjugated with the cryostat. The intensity was detected at 480 nm in the decay curve during TL measurements with a heating rate of 1 K/min. In addition, prior to these measurements, the ZrO_2_ sample was excited at 280 nm, corresponding to the PL excitation peak, for 1 h. The wavelengths for PL and PL excitation were selected according to a previous study^9^. An NIR-laser diode (808 nm, 90 mW) was installed on the cryostat, and pulsed light was generated by combining a function generator with the power supply unit. The waveform was a square wave, with a pulse width of 0.5 s.

## Data avairability

The data and material used and/or analysed during this study are available from the corresponding author on reasonable request.
